# Accuracy and reliability of cetacean cranial measurements using computed tomography three dimensional volume rendered images

**DOI:** 10.1371/journal.pone.0174215

**Published:** 2017-03-22

**Authors:** Adams Hei Long Yuen, Henry Chun Lok Tsui, Brian Chin Wing Kot

**Affiliations:** 1 School of Medical and Health Sciences, Tung Wah College, Homantin, Kowloon, Hong Kong SAR, China; 2 Department of Applied Biology and Chemical Technology, The Hong Kong Polytechnic University, Hunghom, Kowloon, Hong Kong SAR, China; New York Institute of Technology, UNITED STATES

## Abstract

Computed tomography (CT) has become more readily available for post-mortem examination, offering an alternative to cetacean cranial measurements obtained manually. Measurement error may result in possible variation in cranial morphometric analysis. This study aimed to evaluate the accuracy and reliability of cetacean cranial measurements obtained by CT three-dimensional volume rendered images (3DVRI). CT scans of 9 stranded cetaceans were performed. The acquired images were reconstructed using bone reconstruction algorithms. The reconstructed crania obtained by 3DVRI were visualized after excluding other body structures. Accuracy of cranial measurements obtained by CT 3DVRI was evaluated by comparing with that obtained by manual approach as standard of reference. Reproducibility and repeatability of cranial measurements obtained by CT 3DVRI were evaluated using intraclass correlation coefficient (ICC). The results demonstrated that cranial measurements obtained by CT 3DVRI yielded high accuracy (88.05%– 99.64%). High reproducibility (ICC ranged from 0.897 to 1.000) and repeatability (ICC ranged from 0.919 to 1.000 for operator 1 and ICC range from 0.768 to 1.000 for operator 2) were observed in cranial measurements obtained by CT 3DVRI. Therefore, cranial measurements obtained by CT 3DVRI could be considered as virtual alternative to conventional manual approach. This may help the development of a normative reference for current cranial maturity and discriminant analysis studies in cetaceans.

## Introduction

Osteological specimens, particularly crania, represent ideal structures for the study of evolutionary and geographic variation in vertebrate morphology. In cetaceans, the morphometric assessment of cranial measurements parameters may provide additional information to identify sex, age [1–2], evolutionary history, geographical variation [3–6] and sexual dimorphism [3,5–6]. Cetacean cranial morphometrics often rely on manual measurement [7], requiring extensive time and manpower for skeletal excarnation [8–10]. Bone loss may be subsequently induced as consequence of weathering and improper preparation, hindering cranial landmark identification, and thus the accuracy and reliability of corresponding morphometrics. To precisely interpret structural variation of cetacean crania, methods offering accurate and reliable measurements of anatomical specimens must be available.

Cross-sectional imaging techniques, such as computed tomography (CT), have become more readily available for post-mortem examination. A growing body of anatomical data has become available electronically for pathological and biological profile investigation, offering an alternative to traditional cranial morphometrics of cetaceans. In human medicine, CT three-dimensional volume rendering (3DVR) is a non-invasive image reformation technique offering insight of complex, multi-spatial orientation of bony structures [8–11]. It allows operators to bypass the process of skeletal excarnation [9]. The reconstructed CT three-dimensional volume rendering images (3DVRI) can be sectioned in any plane and rotated in space, allowing three-dimensional (3D) insight into the anatomy of the cranium [12]. In humans, Stull and his team [8] demonstrated low average percentage difference between dried bone, the CT 3DVRI of bone before excarnation, and CT 3DVRI of bone after excarnation. In cetaceans, measurement error—either because of uncertainty in landmark identification or alterations in the objects’ true dimensions as a consequence of imaging—could induce possible variability in cranial measurements. To properly utilize this new growing resource, studies must be undertaken to ensure that cetacean cranial measurements obtained by CT 3DVRI are accurate and reliable.

The present study aimed to evaluate the accuracy of cetacean cranial measurements obtained by CT 3DVRI, compared to those obtained by the conventional manual measurement on dried crania as a standard of reference. The present study also examined the inter- and intra-operator variability of cranial measurements obtained by CT 3DVRI.

## Materials and methods

**Fig 1:**
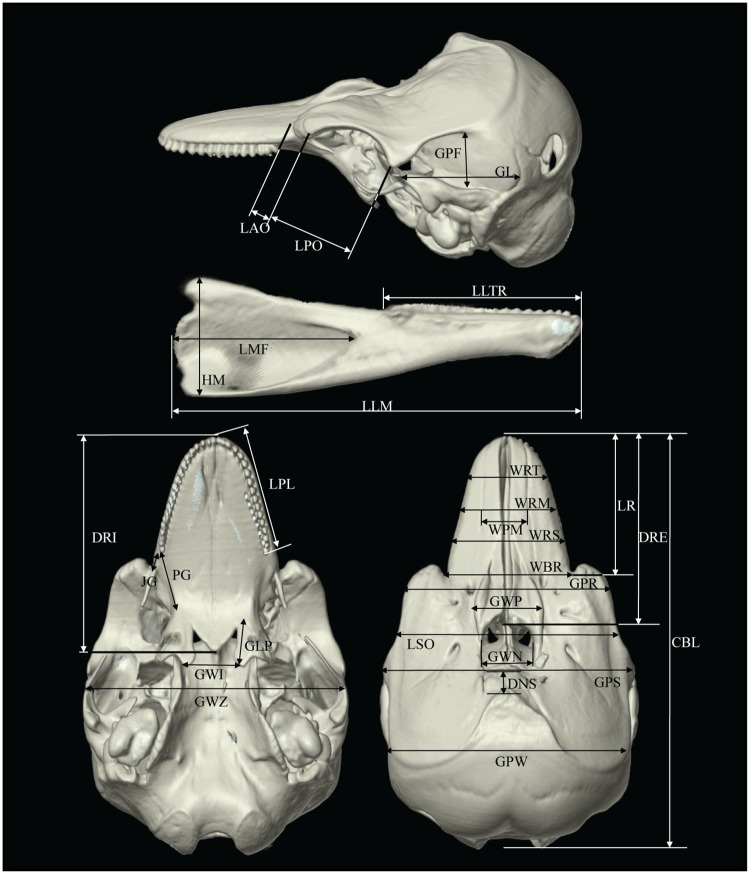
Key to cranial measurements made on CT 3DVRI of *Neophocaena phocaenoides*. For abbreviations, refer to Table 1.

**Fig 2:**
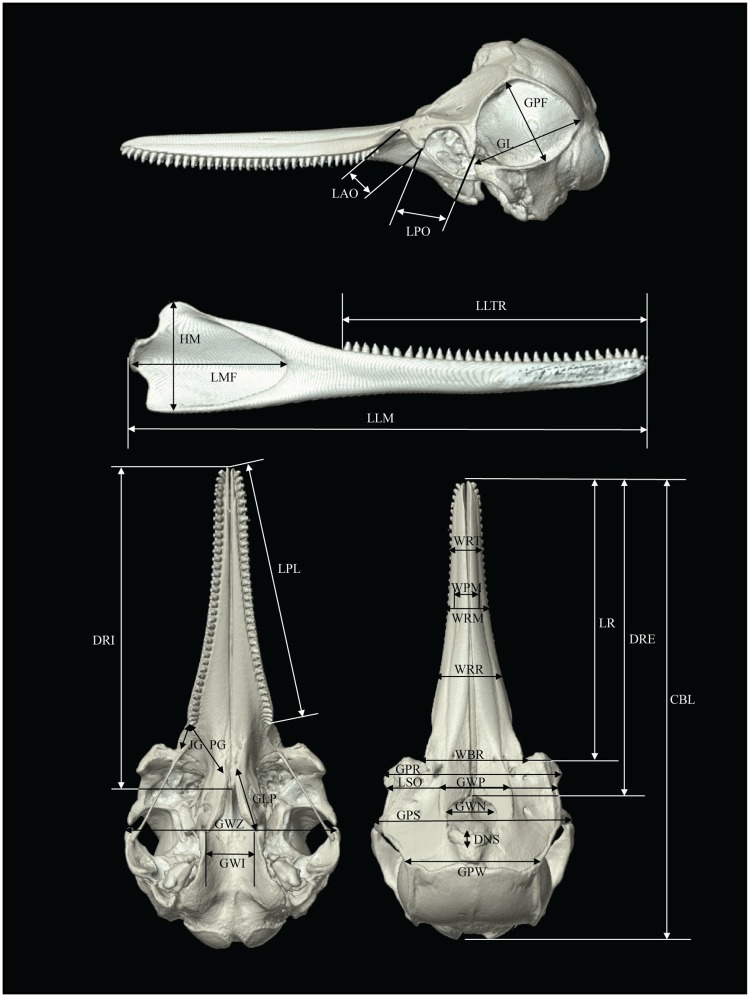
Key to cranial measurements made on CT 3DVRI of *Sousa chinensis*. For abbreviations, refer to Table 1.

A total of 9 cetaceans (8 *Neophocaena phocaenoides* and 1 *Sousa chinensis*) stranded and deceased in the Hong Kong waters in 2012–2015 were included in the present study. Neonates, calves and carcasses with collapsed crania were excluded. The condition of the carcasses was classified using Simthsonian condition codes [13] and ranged from code 2 to 4. Thirty one cranial measurements were collected from each specimen, with reference to the measurement definitions from Yao and her team [7] (Figs 1 and 2, Table 1).

**Table 1:** Descriptions of cetacean cranial measurements in the present study.

Abbreviation	Description of cetacean cranial measurements
CBL	Condylobasal length
LR	Length of rostrum
WBR	Width of rostrum at base
WRR	Width of rostrum at 1/4 length, measured from posterior end
WRM	Width of rostrum at midlength
WPM	Width of premaxillaries at midlength of rostrum
WRT	Width of rostrum at 3/4 length, measured from posterior end
DRE	Distance from tip of rostrum to external nares
DRI	Distance from tip of rostrum to internal nares
GPR	Greatest preorbital width
GPS	Greatest postorbital width
LSO	Least supraorbital width
GWN	Greatest width of external nares
GWZ	Greatest width across zygomatic processes of squamosal
GWP	Greatest width of premaxillaries
GPW	Greatest parietal width, within post temporal fossa
ILB	Internal length of braincase from hindmost limit of occipital condyles to foremost limit of cranial cavity along midline (not illustrated)
GL	Greatest length of left post temporal fossa, measured to external margin of raised suture
GPF	Greatest width of left post temporal fossa at right angles to greatest length
JG	Distance from anterior most end of left jugal to posterior margin of last tooth on upper left rostrum
PG	Distance from anterior most end of left pterygoid to posterior margin of last tooth on upper left rostrum
DNS	Distance from anterior most end of junction between nasals to hindmost point of margin of supraoccipital crest
LPO	Length of left orbit-from apex of preorbital process of frontal to apex of post orbital process
LAO	Length of antorbital process of left lacrimal
GWI	Greatest width of internal nares
GLP	Greatest length of left pterygoid
LPL	Length of upper left tooth row, from hindmost margin of hindmost alveolus to tip of rostrum
LLTR	Length of lower left tooth row
LLM	Greatest length of left ramus
HM	Greatest height of left ramus at right angles to greatest length
LMF	Length of left mandibular fossa, measured to mesial rim of internal surface of condyle

Also see Figs 1 and 2.

All CT scans (2 whole body scans, 1 upper body scan and 6 cranial scans) were performed with a Toshiba 16-row multislice spiral CT scanner Alexion^™^ prior to excarnation. The scans were operated at 100-120kV, 50-170mA, and 1mm slice thickness. Scan field of view (sFOV) was ranged from 15.3cm to 43.2cm. The acquired data sets were reconstructed in 3DVRI using the bone reconstruction algorithm of the inbuilt software (Alexion V4.86ER003). The reconstructed crania obtained by 3DVRI were visualised after excluding other body structures. Two operators (AY and BK) took the cranial measurements obtained by CT 3DVRI in different orientations using the rotation function in the computer graphics system [14]. Cranial measurements obtained by CT 3DVRI were collected by 2 operators (AY and BK) twice. To avoid recall bias, there was a time interval of at least 30 minutes between measurements on the same cranium. Each operator was blinded to the results of the other operator. Operator 1 (AY) had about 2 years of experience in CT scanning and cetacean cranial morphometrics, whereas operator 2 (BK) had more than 4 years of experience.

Following CT scanning, excarnation was performed, removing soft tissue from the crania. They were then air dried at room temperature before proceeding with manual measurement. All manual measurements were taken by AY, thereby eliminating the potential problem of inter-operator bias. Manual measurements were made by using 15cm, 30cm, and 60cm calipers. All measurements were taken to the nearest 0.5mm.

To evaluate the accuracy of the cranial measurements obtained by CT 3DVRI, the measurements taken by AY on 3DVRI were compared to those obtained by the conventional manual approach. The absolute percentage error was calculated. The absolute percentage error of each cranial measurement obtained by CT 3DVRI was defined as the percentage difference between cranial measurement obtained by CT 3DVRI and cranial measurement of dried cranium:

Absolute percentage error (%)=|Cranial measurements obtained by manual approach −Cranial measurements obtained by CT 3DVRI| × 100% ÷(Cranial measurements obtained by manual approach)

The accuracy of the cranial measurements obtained by CT 3DVRI was defined as:

Accuracy (%) = 1 − absolute percentage error

To analyze the intra-operator variability (repeatability) and inter-operator (reproducibility) variability of cranial measurements obtained by CT 3DVRI, intraclass correlation coefficient (ICC) and 95% confidence intervals (C.I.) were used. An ICC > 0.7 was used to indicate sufficient general reliability of the measurements [15–16]. All statistical analyses were performed with the use of SPSS 23.0 software (Statistical Package for the Social Sciences Inc., Chicago, Illinois 60606, USA).

All procedures in this study were reviewed and approved by the Agriculture, Fisheries and Conservation Department of Hong Kong Special Administrative Region [AF GR CON 09/68 PT.9].

## Results

In comparing cetacean cranial measurements obtained by CT 3DVRI with those obtained by manual measurement (Table 2), the mean percentage errors ranged from 0.36% to 11.95%, indicating that measurements obtained by 3DVRI yielded high accuracy (ranging from 88.05% to 99.64%).

**Table 2:** Accuracy of cetacean cranial measurements obtained by CT 3DVRI, using cranial measurements obtained by conventional manual approach as standard of reference.

Cranial measurements	Mean Absolute Error (%)	Mean Measurement Accuracy (%)
CBL	0.36	99.64
LR	3.96	96.04
WBR	1.22	98.78
WRR	1.46	98.54
WRM	3.02	96.98
WPM	6.57	93.43
WRT	3.24	96.76
DRE	2.11	97.89
DRI	1.44	98.56
GPR	1.39	98.61
GPS	1.04	98.96
LSO	1.12	98.88
GWN	2.90	97.10
GWZ	2.08	97.92
GWP	3.87	96.13
GPW	1.71	98.29
ILB	1.70	98.30
GL	3.41	96.59
GPF	5.32	94.68
JG	7.90	92.10
PG	4.71	95.29
DNS	7.46	92.54
LPO	2.21	97.79
LAO	11.95	88.05
GWI	8.14	91.86
GLP	8.88	91.12
LPL	2.50	97.50
LLTR	2.16	97.84
LLM	1.26	98.74
HM	2.38	97.62
LMF	2.57	97.43

For abbreviations, refer to Table 1.

The repeatability of 31 cetacean cranial measurements obtained by CT 3DVRI was shown in Table 3. The ICC values for operator 1 varied from 0.919 to 1.000 with 95% C.I. ranging from 0.685 to 1.000, indicating repeatability of 91.9% to 100%. The ICC values for operator 2 varied from 0.768 to 1.000 with 95% C.I. ranging from 0.265 to 1.000, indicating repeatability of 76.8% to 100%. Overall, results demonstrated that cetacean cranial measurements obtained by CT 3DVRI yielded a high repeatability.

**Table 3:** Intra-operator (repeatability) variability of cetacean cranial measurements obtained by CT 3DVRI.

Cranial measurements	Operator 1		Operator 2	
	ICC (3,1)	95% C.I. of ICC (Lower-Upper)	ICC (3,1)	95% C.I. of ICC (Lower-Upper)
CBL	1.000	1.000–1.000	1.000	1.000–1.000
LR	1.000	0.999–1.000	1.000	0.999–1.000
WBR	0.994	0.972–0.999	0.995	0.980–0.999
WRR	0.978	0.905–0.995	0.958	0.828–0.990
WRM	0.937	0.749–0.985	0.963	0.846–0.992
WPM	0.919	0.685–0.981	0.870	0.530–0.969
WRT	0.956	0.819–0.990	0.982	0.924–0.996
DRE	1.000	0.999–1.000	1.000	1.000–1.000
DRI	1.000	1.000–1.000	1.000	1.000–1.000
GPR	0.998	0.993–1.000	0.998	0.991–1.000
GPS	1.000	0.999–1.000	1.000	1.000–1.000
LSO	0.999	0.995–1.000	0.999	0.997–1.000
GWN	0.994	0.975–0.999	0.995	0.979–0.999
GWZ	0.998	0.991–1.000	0.998	0.990–0.999
GWP	0.996	0.982–0.999	0.995	0.977–0.999
GPW	0.995	0.979–0.999	0.975	0.893–0.994
ILB	0.989	0.954–0.998	0.989	0.954–0.998
GL	0.989	0.952–0.998	0.995	0.979–0.999
GPF	0.997	0.987–0.999	0.997	0.985–0.999
JG	0.992	0.965–0.998	0.969	0.869–0.993
PG	0.960	0.833–0.991	0.984	0.931–0.996
DNS	0.918	0.680–0.981	0.768	0.265–0.943
LPO	0.984	0.931–0.996	0.941	0.763–0.986
LAO	0.973	0.884–0.994	0.983	0.925–0.996
GWI	0.994	0.972–0.999	0.994	0.975–0.999
GLP	0.994	0.973–0.999	0.990	0.955–0.998
LPL	1.000	0.999–1.000	1.000	0.999–1.000
LLTR	1.000	0.999–1.000	1.000	1.000–1.000
LLM	1.000	1.000–1.000	1.000	1.000–1.000
HM	0.999	0.994–1.000	0.999	0.995–1.000
LMF	0.998	0.990–0.999	0.999	0.998–1.000

For abbreviations, refer to Table 1.

The reproducibility of 31 cetacean cranial measurements obtained by CT 3DVRI was shown in Table 4. The ICC values were ranging from 0.897 to 1.000 with 95% C.I. ranging from 0.608 to 1.000, indicating reproducibility of 89.7% to 100%. Results demonstrated that cetacean cranial measurements obtained by CT 3DVRI yielded a high reproducibility.

**Table 4:** Inter-operator (reproducibility) variability of cetacean cranial measurements obtained by CT 3DVRI.

Cranial measurements	ICC (2,1)	95% C.I. of ICC (Lower-Upper)
CBL	1.000	0.999–1.000
LR	1.000	0.999–1.000
WBR	0.994	0.972–0.999
WRR	0.942	0.778–0.987
WRM	0.962	0.842–0.991
WPM	0.897	0.608–0.976
WRT	0.976	0.871–0.992
DRE	1.000	1.000–1.000
DRI	1.000	1.000–1.000
GPR	0.998	0.991–1.000
GPS	1.000	1.000–1.000
LSO	0.998	0.994–1.000
GWN	0.991	0.958–0.998
GWZ	0.999	0.996–1.000
GWP	0.996	0.975–0.999
GPW	0.981	0.920–0.996
ILB	0.991	0.960–0.998
GL	0.999	0.995–1.000
GPF	0.997	0.988–0.999
JG	0.934	0.698–0.985
PG	0.948	0.786–0.988
DNS	0.917	0.685–0.981
LPO	0.945	0.664–0.988
LAO	0.975	0.899–0.994
GWI	0.994	0.950–0.999
GLP	0.908	0.651–0.978
LPL	1.000	0.998–1.000
LLTR	1.000	1.000–1.000
LLM	1.000	1.000–1.000
HM	0.999	0.997–1.000
LMF	0.997	0.987–0.999

For abbreviations, refer to Table 1.

## Discussion

CT scanning was widely used for evaluation of osteological structures because of its documented high spatial resolution, contrast resolution, and signal-to-noise ratio. The image quality, measurement accuracy and reliability of 3DVRI were largely depended on the slice thickness, sFOV and reconstruction algorithm [17]. The data acquired in the present study was with slice thickness of 1 mm. Slice thicknesses greater than 1.25 mm may require more interpolation between slices when rendering CT 3DVRI, which results in a loss of fidelity of the representative anatomy [18]. Such data loss can lead to inaccuracies in cetacean cranial measurements. Bone reconstruction algorithm with minimized sFOV was also used in the present study to produce adequate 3DVRI resolution for accurate cranial measurements.

The results of the present study show that cetacean cranial measurements obtained by CT 3DVRI are highly accurate (88.0–99.6%) when compared with cranial measurements obtained by manual measurement. Of the 31 cranial measurements included in the present study, relatively lower accuracy (88%) was observed in length of antorbital process of left lacrimal (LAO). The cause of the slight deviation was related to 3DVR software interpolates data between scan slices.

The present study also demonstrated high repeatability and reproducibility in cranial measurements obtained by CT 3DVRI. Among the 31 cranial measurements, the lowest repeatability was observed in width of premaxillaries at midlength of rostrum (WPM) and distance from anterior most end of junction between nasals to hindmost point of margin of supraoccipital crest (DNS), while the lowest reproducibility was observed in length of left orbit-from apex of preorbital process of frontal to apex of post orbital process (LPO), distance from anterior most end of left jugal to posterior margin of last tooth on upper left rostrum (JG), greatest length of left pterygoid (GLP), WPM and DNS. Measurements of WPM, DNS, LPO and JG might be subject to the effect of interpolation between slices when rendering CT 3DVRI, which resulted in loss of cranial landmarks or sutures definition, affecting precision of caliper placement. Variation in GLP measurement may be accounted by the relatively low bone density in cetacean pterygoid hamulus, which leads to the failure in reconstructing the intact pterygoid as a result of inability to display actual Hounsfield units during conversion from CT number to electron density. Nonetheless, most cranial characters presented in 3DVRI were recognizable and thus, cranial measurements were considered to be accurate and reliable.

CT 3DVRI data set was recorded in Digital Imaging and Communication in Medicine (DICOM) format in the present study. DICOM data sets can be permanently stored in Picture Archiving and Communication System, which can be recalled at will to the same specimen if additional information is required. Its digitally transferable feature can also facilitate second opinion by other professionals or institutes worldwide even if the specimens cannot not be physically provided or available.

CT scanning of cetaceans’ cranium can be a valuable resource to identify and document osteological structures, as well as soft tissues that were not preserved in osteological collections. Spatial relationship between specific organs, soft tissues and bones can be selectively investigated in situ in their true dimensions and positions [19–20]. CT 3DVRI could be a valuable approach for obtaining morphological information from cetaceans. Since cross-sectional imaging technologies are now more readily available in studies of animals’ taxonomy and life history, there are increasing collaboration between museum and imaging centres to digitalize specimens [21–22]. Digitalized specimens would allow for investigations of anatomical characters and might increase the accessibility of phenotypical data [21–22].

To conclude, the results of the present study demonstrate that cetacean cranial measurements obtained by CT 3DVRI were accurate and reliable. CT 3DVRI could be considered a promising alternative to traditional manual cranial measurements in providing information for biological profiles investigation in cetaceans.
